# Visual and noxious electrical stimulus-evoked membrane-potential responses in anterior cingulate cortical neurons

**DOI:** 10.1186/s13041-016-0262-y

**Published:** 2016-09-01

**Authors:** Li-qing Ma, Li Ning, Zhiru Wang, Ying-wei Wang

**Affiliations:** 1 Department of Anesthesiology, Huashan Hospital, Fudan University, No. 12 Wulumuqi Road, Shanghai, 200040 China; 2 Institute and Key Laboratory of Brain Functional Genomics of Shanghai and Chinese Ministry of Education, Shanghai Key Laboratory of Brain Functional Genomics, School of Life Sciences, East China Normal University, No. 3663 Zhongshang Road, Shanghai, 200062 China; 3 Department of Anesthesiology, Xinhua Hospital, Shanghai Jiaotong University School of Medicine, Shanghai, 200092 China

**Keywords:** Anterior cingulate cortex, Visual stimulus, Visual thalamus, Visual cortex, Pain

## Abstract

Anterior cingulate cortex (ACC) is known to participate in numerous brain functions, such as memory storage, emotion, attention, as well as perception of acute and chronic pain. ACC-dependent brain functions often rely on ACC processing of various forms of environmental information. To understand the neural basis of ACC functions, previous studies have investigated ACC responses to environmental stimulation, particularly complex sensory stimuli as well as award and aversive stimuli, but this issue remains to be further clarified. Here, by performing whole-cell recording in vivo in anaesthetized adult rats, we examined membrane-potential (MP) responses of layer II/III ACC neurons that were evoked by a brief flash of visual stimulation and pain-related electrical stimulation delivered to hind paws. We found that ~54 and ~81 % ACC neurons exhibited excitatory MP responses, subthreshold or suprathreshold, to the visual stimulus and the electrical stimulus, respectively, with no cell showing inhibitory MP responses. We further found that the visually evoked ACC response could be greatly diminished by local lidocaine infusion in the visual thalamus, and only their temporal patterns but not amplitudes could be changed by large-scale visual cortical lesions. Our in vivo whole-cell recording data characterized in ACC neurons a visually evoked response, which was largely dependent on the visual thalamus but not visual cortex, as well as a noxious electrical stimulus-evoked response. These findings may provide potential mechanisms that are used for ACC functions on the basis of sensory information processing.

## Introduction

As a major part of brain’s limbic systems, the anterior cingulate cortex (ACC) is involved in numerous brain functions, including learning and memory [1–4], emotion [5–8], attention [1, 9, 10], as well as perception of acute and chronic pain [11–14]. With respect to the neural basis of ACC functions, anatomical studies have shown that ACC is reciprocally connected with a variety of subcortical and cortical regions, including the lateral prefrontal cortex, parietal cortex, motor cortex, nucleus accumbens, amygdala, and hippocampus [15–20]. Consistently, neurophysiological studies have shown that ACC neurons can respond to multiple forms of environmental stimulation, particularly the responsiveness to complex and noxious stimuli, as indicated by the activity of ACC neurons in association with reward [21–23] and aversive stimuli [7, 24–26] as well as attention [1, 9, 10, 27]. To further understand the neural mechanism accounting for ACC functions, ACC responses to sensory stimulation remain to be further elucidated. Using in vivo whole-cell recording from anaesthetized adult rats, we examined the response evoked by a brief flash of visual stimulation in layer II/III ACC neurons, and compared the responses with those elicited by a painful stimulus. We found that a large amount (~54 %) of ACC neurons exhibited excitatory membrane-potential (MP) responses to the flash stimulus, and these responses were further found to be largely dependent on the visual thalamus rather than the visual cortex. In a larger population (81.25 %) of ACC neurons, we also observed excitatory MP responses to a pain-related DC electrical stimulus that was delivered to hind paws. The characterized responsiveness of ACC neurons to simple sensory stimulation and pain-related stimulation may provide further understanding of the mechanism for ACC-dependent brain functions.

## Methods

### Surgery and preparation

Adult Sprague–Dawley rats (male, 10–14 weeks old, 300–380 g) were used in this study. All animal procedures were performed in accordance to Animal Care and Use Committee of East China Normal University, where the experiments were performed. Experimental procedures were similar to that described previously [28]. In brief, animals were initially anaesthetized with pentobarbital (80 mg/kg; i.p.). After tracheotomy, the head was restrained in a stereotaxic apparatus (David Kopf Instr.) with the body temperature maintained at 37.3–37.8 °C. For recording, a small craniotomy (~2 mm in diameter) was made above the left cortex, and a small piece of dura mater was carefully removed. Eyes were fixed to metal rings and irrigated with saline. The anaesthesia level was maintained with supplementary injection of pentobarbital (16―20 mg/kg/h; i.p.). Electrophysiological recordings were conducted at a light anaesthesia level just below the threshold of body movements consisting of licking or scratching.

### In vivo electrophysiological recording and stimulation

Patch pipettes with tip opening of 2.5–3.0 μm were pulled with a vertical puller (PC-10, Narishige). Internal solution contained (in mM) 136.5 K-Gluconate, 17.5 KCl, 9.0 NaCl, 1.0 MgCl_2_, 10.0 HEPES, 0.2 EGTA, and Amphotericin B (0.5 mg/ml), and small amounts (0.5–0.8 mg/ml) of glass beads (5–15 μm in diameter; Polysciences, Inc.) were included to prevent the entry of the precipitates of Amphotericin-containing solution in pipette opens. The pH of the internal solution was adjusted to 7.3. Patch pipettes were advanced in the brain with a motor-driven manipulator (Siskiyou MMX7630, Siskiyou Corp.), during which a positive pressure was applied into the pipettes. Signals were acquired with a patch-clamp amplifier (Axopatch 200B, Axon Instr.), and sampled at 5 or 10 kHz by a data acquisition card (Digidata 1440, Axon Instr.) with 1, 2, or 5 kHz low-pass filtering. ACC recordings were made from neurons with a relatively slow action-potential half-width (mean ± SD, 1.43 ± 0.2 ms; measured from the spontaneous firing of 27 randomly selected neurons), which were putatively pyramidal cells [29, 30]. That the whole-cell recordings were made from ACC and the visual cortex was assessed by the stereotaxic coordinates of recording sites (from Bregma, 1.2–2.8 mm anterior, 0.2–0.6 mm lateral, and 1.5–2.0 mm beneath the cortical surface for ACC recordings; 6–8 mm posterior and 3–4.5 mm lateral for visual cortical recordings), which were measured for each recording. Histological staining including that for neuronal morphology was performed in some experiments to confirm the recording site, as shown in our previous study [28]. For extracellular recording, glass pipettes (filled with saline) with tip opening of 5–8 μm were used.

For visual stimulation, two LED screens (6 × 6 cm) were placed at a distance of 1.5–2 cm from eyes. Flash stimuli (100-ms duration) with the luminance of 180–200 cd m^-2^ (background, 2–4 cd m^−2^) was applied to bilateral eyes. For electrical stimulation, two metal wires were attached to the two hind paws (as illustrated in Fig. 9a), and DC currents (2 mA, 2 s) were produced by a pulse generator (Master-8; A.M.P.I.) through a stimulus isolator (ISO-Flex; A.M.P.I.). For each cell, 100–200 repeats of response measurements were conducted.

### Lidocaine infusion and V1 lesions

For LGN infusion, guide cannulas (28 gauge) were bilaterally implanted in the brain (tips from Bregma: 4–4.5 mm lateral and 4.0–4.5 mm posterior; 4.5–5 mm beneath the cortical surface) and fixed on the skull with dental acrylic. Internal injection cannulas (32 gauge) inserted in guide cannulas were used for drug application. For V1 infusion, glass pipettes with tip opening of ~50 μm were used, which were placed at two ipsilateral V1 sites and fixed with manipulators (tips from Bregma: 4 mm lateral and 6 mm posterior; 3.5 mm lateral and 8 mm posterior; both were 0.4–0.6 mm beneath the cortical surface). V1 lesions were performed by repeated removal of the cortical tissue with the use of a 26-gauge needle and fine forceps.

### Nissl staining

After electrophysiological recording, the V1-lesioned rat was perfused with 4 % paraformaldehyde (PFA) in 0.1 M PBS. Brains were immediately removed and fixed in PFA for 2 days at 4 °C, which was followed by cryoprotection in 20 and 30 % sucrose. Fifty-μm thick slices were cut with a cryostat (Leica CM1850, Leica Corp.) and stained with cresyl violet for 5–6 min.

### Data analysis and statistics

**Fig. 1:**
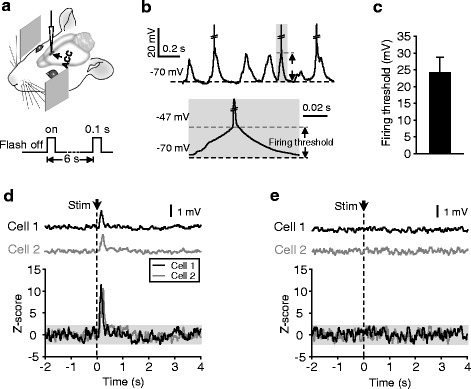
Excitatory responses of ACC neurons to flash stimuli. **a** Schematic of recording and visual stimulation. **b** Top, spontaneous firing and MP changes for an example cell; action potentials are truncated; black dash lines, resting potentials; gray dash line on shadowed area, firing threshold (defined as the MP value at which dV/dt > 10 V/s; see Methods). Bottom, shadowed area at (top) shown with high temporal resolution. **c** Firing threshold (from resting potentials; as illustrated in (**b**)) in the spontaneous events measured from 58 randomly selected neurons. **d** and **e** Average MP changes in response to the flash stimulus (top) and the corresponding Z-score values (bottom; the −2 ≤ values ≤ 2 are shadowed) (**e**). Time 0 is the time point of stimulus onset (arrows and dash lines)

For whole-cell recording, a liquid junction potential of −13 mV has been corrected. Recordings with resting potentials between −65 mV and −80 mV (−73 ± 5.5 mV; mean ± SD) were included for further analysis, which had a membrane resistance of 65 ± 14 MΩ (mean ± SD), and a series resistance (which was not compensated) of 53 ± 13 MΩ. In 33 recordings, we monitored input resistances by applying a hyperpolarizing pulse (80–120 pA; 200 ms; 30-s intervals), and found a change of input resistances that was smaller than 25 % in 31 recordings during the whole-period of measurements. Overshooting spikes could be usually observed in the recordings. To average MP responses, spikes were removed from each trial with cutoff amplitudes at firing thresholds (defined as the MP value at which dV/dt > 10 V/s). In the average MP responses, baseline noises were 0.35 ± 0.13 (mean ± SD). MP and SR responses were defined as Z-score values of the peak amplitudes > 2 (with reference to SD in baseline noises before stimulation; as shown for two representative recordings in Fig. 1d, e). The presence or absence of responses that was defined on this basis also met the criterion of *P* < 0.05 or not (Wilcoxon signed rank test). Onset latencies and durations were inspected according to 2-fold-s.d. amplitudes.

Unless otherwise specified, statistical significance was determined using Mann- Whitney *U* test, and average values are presented as mean ± SEM.

## Results

### Visually evoked excitatory responses in layer II/III neurons of ACC

**Fig. 2:**
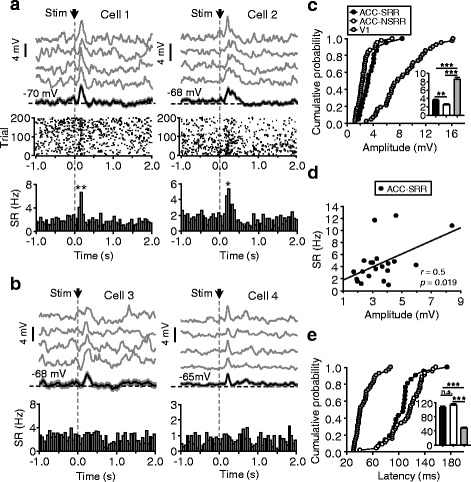
Properties of ACC and V1 responses to flash stimuli. **a** Two example recordings showing both MP and SR responses. Top, average MP responses; gray and black (with shadowed areas that indicate SEM) traces represent the averages of 50 consecutive and all (200) trials, respectively. Black dash line, mean MP. Middle and bottom, raster plots (middle) and peristimulus time histograms (PSTHs; bin width, 50 ms) (bottom) of SR responses. Arrows and gray dash lines, time points of stimulus onset. **b** Two example cells with MP responses (top) but no SR responses (PSTHs shown at bottom). **c** Cumulative distribution plots for the peak amplitudes of MP responses recorded from the ACC neurons that exhibited SR responses (ACC-SRR; *n* = 21) and not (ACC-NSRR; *n* = 44) and from V1 neurons (*n* = 42; recordings with and without SR responses were both included). Histograms in insets: mean ± SEM. **d** For ACC neurons showing SR responses, peak amplitudes of MP responses plotted against SR; line, linear regression fits. **e** Same as (**c**), for response latencies. *p < 0.05, **p < 0.01

We performed perforated whole-cell recording from layer II/III neurons of ACC (as illustrated in Fig. 1a) in lightly anaesthetized adult rats. Neuronal activity was monitored at resting potentials in current-clamp mode (without applying injection currents). In the absence of sensory stimuli, ACC neurons exhibited spontaneously occurring MP oscillations (2.4 ± 0.44 (SD) Hz), which were usually accompanied by neuronal firing (1.4 ± 1.1 (SD) Hz) (see Fig. 1b for an example cell), and a firing threshold (from resting potentials) of 24 ± 4.7 (SD) mV was observed in our recordings (Fig. 1c). To investigate sensory stimulus-evoked ACC responses, we presented a brief flash (100-ms duration) of visual stimulation to bilateral eyes and measured MP and spike-rate (SR) responses. In a total of 120 recordings obtained from layer II/III of ACC, 65 (54.2 %) cells displayed excitatory responses, and no cells showed inhibitory responses (see Discussion), as shown in Fig. 1d, e for the averaged traces of two responsive cells and two unresponsive cells. In the 65 responsive cells, 21 (17.5 % of all the ACC recordings) showed SR increase (see Fig. 2a, b for 4 example recordings and Table 1 for the summarized results).

**Table 1:** Fractions of ACC neurons responsive to flash stimuli or electrical stimuli

N/total *N* (%)	Responsive		Unresponsive
	SRR	NSRR	
F-Stim	21/120 (17.5 %)	44/120 (36.7 %)	55/120 (45.8 %)
F-Stim & V1 lesioned	8/50 (16 %)	14/50 (28 %)	28/50 (56 %)
E-Stim	14/32 (43.75 %)	12/32 (37.5 %)	6/32 (18.75 %)

F-Stim represents the measurement of flash stimulus-evoked responses (experiments summarized in Fig. 2; the data obtained in the following experiments with lidocaine infusion in LGN or V1 were not included); E-Stim represents electrical stimulus-evoked responses. SRR and NSRR represent the cells showing SR responses and not, respectively

Compared with the excitatory responses evoked by the same flash in the primary visual cortex (V1; all V1 neurons were found to be responsive to the same flash, and the majority of the cells showed excitatory responses), the excitatory MP responses of ACC neurons were much smaller (V1, 8.5 ± 0.5 mV; ACC, 2.9 ± 0.2 mV; *p* < 0.001) (Fig. 2c). In ACC neurons, the MP responses accompanied by SR responses were significantly larger than those without SR responses (3.5 ± 0.3 mV and 2.6 ± 0.2 mV for the former and latter groups, respectively; *p* = 0.004) (Fig. 2c). In the elicited ACC spiking, a SR ranging from 1.3 to 7.7 Hz (4.5 ± 0.7 Hz) was observed, and the SRs were positively correlated with their MP response amplitudes (Fig. 2d; *r* = 0.5, *p* = 0.019). On the other side, a slight but not significant difference was found between the onset latencies of MP responses of the ACC neurons that exhibited SR responses and not (105 ± 5 ms and 113 ± 4 ms for the former and latter groups, respectively, *p* = 0.081), both of which were much longer than V1 responses (48 ± 2 ms) (Fig. 2e).

### Visually evoked ACC responses originate from the visual thalamus

**Fig. 3:**
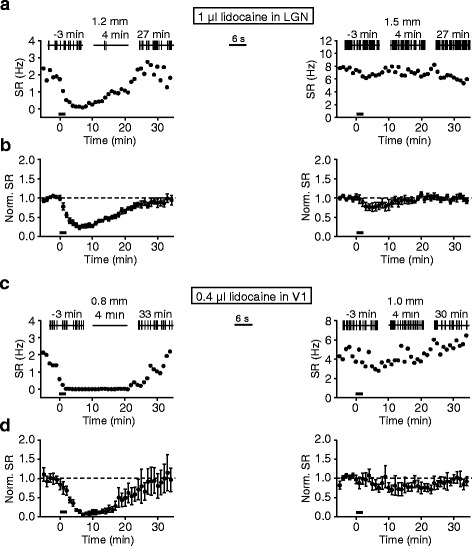
Distance estimation of blocking effects on neuronal firing by LGN- and V1-lidocaine infusion. **a** Examples of SR changes caused by 1 μl lidocaine infusion in LGN, which were measured at the distances of 1.2 mm (left) and 1.5 mm (right) from infusion sites; drugs were applied during 0–2 min (indicated by thick bars); insets at top, representative SRs (time points of firing are indicated by vertical bars) before and after drug infusion. **b** Summary of all experiments shown in (**a**) (*n* = 13 and 10 for recordings at 1.2 mm and 1.5 mm, respectively); data were normalized to mean SRs measured within 0–5 min before drug application. **c** and **d** Same as (**a**) and (**b**), but for V1-lidocaine infusion, in which 0.4 μl lidocaine was applied for 2 min and SR recordings were obtained at the distances of 0.8 mm and 1.0 mm from infusion sites (*n* = 10 and 9 for recordings at 0.8 mm and 1.0 mm, respectively)

We next determined whether the ACC responses to flash stimuli originated from the classical visual pathway from the retina to visual thalamus. For this purpose, lidocaine (2 %, 1 μl) was bilaterally infused into the central site of the lateral geniculate nucleus (LGN) (see Methods for locations). As demonstrated by our extracellular recordings in the thalamus, this treatment could block neuronal firing occurring within ~1.5 mm from infusion sites (see Fig. 3a, b). Thus, a local region of LGN and a small part of surrounding (extra-LGN) regions were exposed to the drug treatment.

**Fig. 4:**
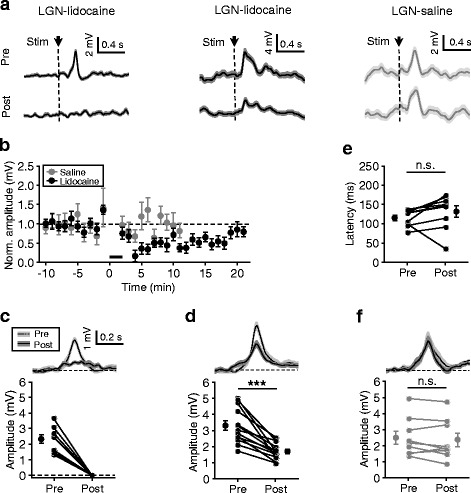
Blocking effects of local LGN-lidocaine infusion on ACC responses to flash stimuli. **a** Example data showing average pre- and post-infusion MP responses, in which the responses were totally (left) or partially (middle) blocked by lidocaine and were unchanged by saline (right). In these experiments, 1 μl lidocaine or saline was bilaterally infused in LGN, and, for each infusion site, application was the same as the experiments shown in Fig. 3 (**a** and **b**). **b** Summary of all experiments shown in (**a**) (*n* = 22 for lidocaine group, in which, all cells were recorded for at least 10 min after drug infusion and 16 cells for ~22 min; *n* = 9 for saline group); data were normalized to the amplitude of pre-infusion responses; thick bars represent drug application. **c** and **d** For cells with responses totally (**c**) (*n* = 9) or partially (**d**) (*n* = 13) blocked by lidocaine treatment. Top, pre- and post-infusion responses averaged across population data in reference to the peak time of pre-infusion responses. Bottom, plots for pre- and post-infusion response amplitudes of all individual recordings; for no significant responses, corresponding values were plotted at 0 mV; dots connected by lines, measurements from the same cells; dots with bars, mean ± SEM. **e** For the cells shown in (**d**) but with post-infusion responses exceeding 1.5 mV (*n* = 9), plots for their onset latencies. **f** Same in (**c** and **d**), for all individual recordings of saline controls (*n* = 9). *** *p* < 0.001, n.s., not significant

By recording ACC neurons, we found that their MP responses to flash stimuli could be totally or partially blocked by such lidocaine application into the LGN, as shown by the average responses measured before (pre-) and after (post-) drug infusion for two example recordings (Fig. 4a, left and middle), and this blocking effect could last for ~20 min (see Fig. 4b for the time course plots for response amplitudes of all cells, in which, 22 cells recorded for at least 10 min and 16 cells recorded for ~22 min after drug application). We further selected the measurements obtained within 10 min before and within 10 min after drug infusion to evaluate the extent of the blocking effects. In 9/22 (41 %) neurons, post-infusion responses were found to be totally blocked (i.e., no significant responses detected), and these cells exhibited a pre-infusion responses of 2.3 ± 0.3 mV (Fig. 4c). In the cells showing significant post-infusion responses (*n* = 13), the response amplitudes were greatly reduced (from 3.3 ± 0.3 mV to 1.7 ± 0.1 mV, ~50 % reduction; *p* < 0.001, Wilcoxon signed rank test) (Fig. 4d). Between the onset latencies of the pre- and post-infusion responses, no significant difference was found (Fig. 4e) (*n* = 9; analysis was conducted by including the cells with post-injection responses exceeding 1.5 mV; *p* = 0.098, Wilcoxon signed rank test). As a control experiment, applying saline in LGN via the same procedures did not alter ACC responses (see Fig. 4a, right for an example; see Fig. 4b, f for summary (*n* = 9; *p* = 0.301, Wilcoxon signed rank test)).

In the above experiments using LGN-lidocaine infusion, although a small part of extra-LGN regions could be exposed to the drugs, this extra-LGN effect seems unlikely to account for the reduction of ACC responses, due to the lack of direct influences of these regions on visual transmission, particularly at the classical visual pathway. Thus, the significant reduction of response amplitudes by LGN-lidocaine infusion indicates that the flash-evoked ACC responses arose from the visual thalamus. On the other hand, the retained responses observed with LGN-lidocaine infusion could be attributed to the local nature of drug application in LGN and, as another possibility, arose from other subcortical regions such as superior colliculus [31].

### Visual cortical modulation on the temporal pattern of ACC responses

**Fig. 5:**
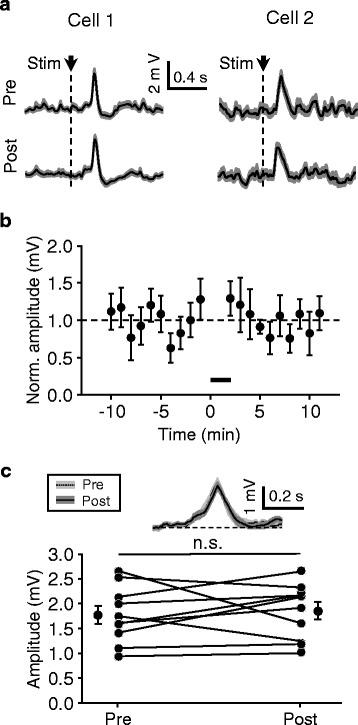
No significant effects of local V1-lidocaine infusion on ACC responses to flash stimuli. **a** Average pre- and post-infusion responses for two examples, in which 0.4 μl lidocaine was applied in two ipsilateral V1 sites, and, for each infusion site, application was conducted via the same procedures as the experiments shown in Fig. 3 (c and d). **b** and **c** Summary of all experiments shown in (**a**) (*n* = 10). Bar in (**b**), drug infusion; traces in (**c**), pre- and post-infusion responses averaged across population data in reference to their peak times

We next examined the possible contribution of the visual cortex to flash-evoked ACC responses by locally infusing lidocaine (2 %, 0.4 μl) into two ipsilateral sites (see Methods for locations). In our observations, this treatment was found to block neuronal firing that occurred within ~1 mm from injection sites (see Fig. 3c, d). To avoid drug diffusion into the lateral ventricle, we placed the tip of infusion pipettes at 0.4–0.6 mm beneath the cortical surface. In contrast to the findings obtained with local LGN-lidocaine treatment, such local V1-lidocaine infusion did not appear to have any effect on the amplitude of flash-evoked ACC responses, as indicated by the averaged pre- and post-infusion responses for two example data (Fig. 5a), the time courses plotted for response amplitudes of all cells (*n* = 10) (Fig. 5b), and the amplitudes of pre- and post-infusion responses plotted for the same cells (Fig. 5c; *p* = 0.49 for comparison, Wilcoxon signed rank test).

**Fig. 6:**
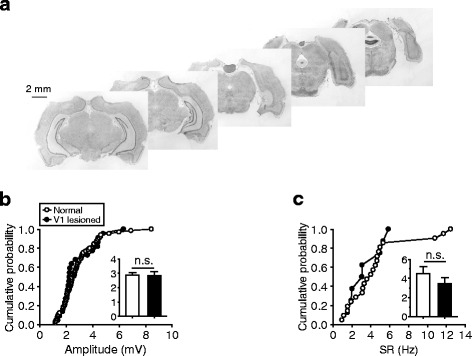
No significant changes in the amplitude of ACC responses by large-scale visual cortical lesions. **a** Representative images of Nissl-stained sections from a rat (section thickness, 50 μm), in which large-scale lesions had been made mechanically in bilateral V1. **b** Amplitudes of MP responses recorded in normal rats (*n* = 65; data are shown in Fig. 2 (**c**)) and in rats receiving V1 lesions (*n* = 22); cells with and without SR responses were included for each group of data. **c** For SR responses recorded in normal (*n* = 21) and lesioned groups (*n* = 8)

That the ACC responses were not changed by lidocaine infusion in V1 could be due to the local nature of drug application. In the following experiments, we mechanically made lesions in the entire, bilateral V1 regions (see Fig. 6a for representative images and Methods) to further investigate the possible involvement of the visual cortex in visual transmission to ACC neurons. In a total of 50 recordings obtained with the V1 lesions, 22 (44 %) neurons exhibited significant excitatory MP responses to the flash stimulus, and 8 (16 %) exhibited significant SR responses (Table 1). Both fractions were similar to those observed in normal rats (*p* = 0.30 by Chi-square test and *p* = 0.8 by Fisher exact test for MP and SR responses, respectively). Again, with respect to response magnitudes, both the MP and SR responses were similar to those observed in normal rats (*p* = 0.77 and for MP and *p* = 0.68 for SR responses) (Fig. 6b, c). Thus, even this bilateral large-scale lesion in the visual cortex could not change the amplitude of visually evoked responses in ACC neurons.

**Fig. 7:**
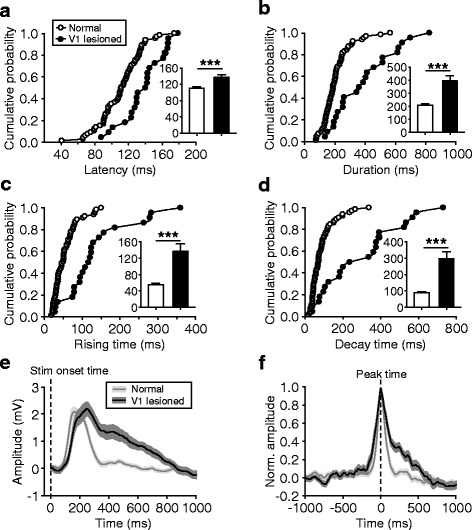
Changes in the temporal profile of ACC responses by large-scale visual cortical lesions. **a**–**d** For the responses of normal and V1-lesioned groups, cumulative distributions and mean values (insets) of the onset latencies (**a**), durations (**b**), rising times (**c**), and decay times (**d**). **e** and **f** Responses averaged across population data in reference to stimulus onset times (**e**) and in reference to response peak times (**f**)

**Fig. 8:**
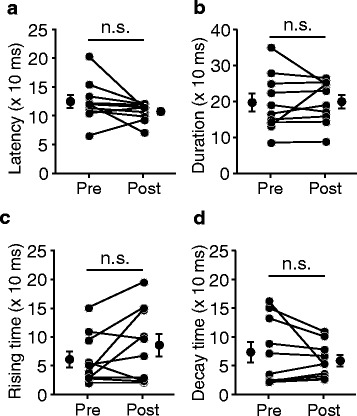
No changes in the temporal profile after local lidocaine infusion in V1. **a**–**d**, For all individual data recorded with local lidocaine treatment in V1 (data shown in Fig. 5), the latencies (**a**), durations (**b**), rising times (**c**), and decay times (**d**) of pre- and post-infusion responses (*n* = 10)

However, unlike the magnitude, the temporal profile of the ACC responses was significantly altered by the V1 lesions. In our analysis, a longer onset latency (138 ± 5 ms) compared with the recordings from normal rats (111 ± 3 ms; data from Table 1) was detected (Fig. 7a*; p* < 0.001), suggesting visual cortical effects to speed up visual information transmission to ACC. In addition, the duration of the responses was also found to be largely increased (from 206 ± 12 ms to 393 ± 42 ms; nearly 1-fold change; *p* < 0.001) (Fig. 7b), and this increase consisted of a longer time in both rising and decay phases (20–80 % peak amplitudes) (Fig. 7c, d). The changes in onset latencies and durations can also be discerned from the responses averaged across population data in reference to stimulus onset times (Fig. 7e) and in reference to response peak times (Fig. 7f). In the further analysis of the data recorded with local V1-lidocaine infusion (experiments shown in Fig. 5), no such changes in the temporal profiles were observed (see Fig. 8 for the comparisons of pre- and post-infusion responses; onset latency, *p* = 0.13, Wilcoxon signed rank test; duration, *p* = 0.63; rising time, *p* = 0.16; decay time, *p* = 0.56), probably because the drug application was of a local nature. These findings indicate that, by recruiting a relatively large neuronal population, the visual cortex may be able to change the temporal profile but not the magnitude of visual responses of ACC neurons, a process that is likely to involve top-down projections from the visual cortex to the visual thalamus [32]. However, the possibility that the V1 lesion (as an acute brain trauma) by itself could change the temporal profile cannot be excluded.

### Noxious electrical stimulus-evoked excitatory responses in layer II/III neurons of ACC

**Fig. 9:**
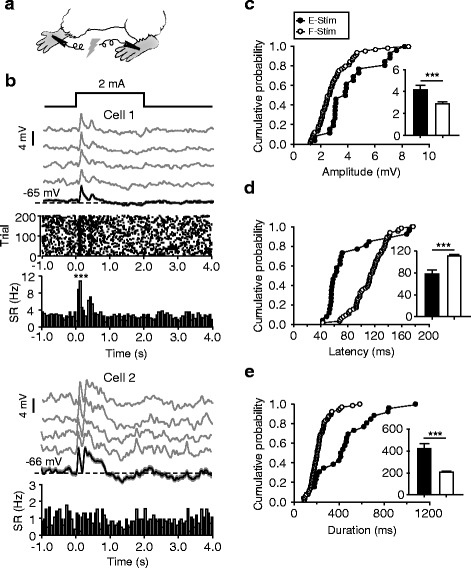
Excitatory responses of ACC neurons to electrical stimuli. **a** Schematic of DC stimulation delivered to hind paws. **b** Two example ACC neurons showing electrical stimulus-evoked MP responses that generated SR responses (Cell 1) and not (Cell 2). Data are presented as in Fig. 2a, **b** inset at top indicates the application of the DC electrical stimulus (2 mA, 2 s). **c**–**e** For the MP responses evoked by electrical stimuli (E-Stim, *n* = 26) and flash stimuli (F-Stim, *n* = 65; experiments shown in Fig. 2), cumulative distribution plots for peak amplitudes (**c**), onset latencies (**d**), and durations (**e**). Histograms in insets: mean ± SEM. ***p < 0.001

Previous studies have shown that ACC neurons can respond to noxious or painful stimulation [24, 26, 30, 33], and this responsiveness is also attributed to a neural basis for ACC-dependent brain functions. In this study, we have also investigated MP responses of ACC neurons to a DC electrical stimulus (2 mA, 2 s), which was delivered to hind paws (as illustrated in Fig. 9a), and compared these responses with those evoked by the flash visual stimulus. In a large population (26/32; 81.25 %) of ACC neurons recorded in layer II/III, we observed significant excitatory MP responses to the electrical stimulus, and 14 cells (43.75 % of all recordings) displayed SR responses (Table 1; see Fig. 9b for the responses of two example cells). Similar to that observed with visual stimuli, no cell showed inhibitory MP responses to electrical stimuli. As compared with the excitatory MP responses to the visual stimulus (data summarized in Fig. 2), electrical stimulus-evoked MP responses in ACC neurons exhibited a significantly larger amplitude (4.2 ± 0.4 mV, *p* < 0.001; Fig. 9c) as well as both a shorter onset latency (78 ± 8 ms, *p* < 0.001; Fig. 9d) and duration (420 ± 49 ms, *p* < 0.001; Fig. 9e).

## Discussion

Sensory information processing in ACC networks is critical for ACC-related brain functions. Using in vivo whole-cell recording, we have investigated MP responses of layer II/III ACC neurons to sensory stimulation. We first found that ~54 % ACC neurons exhibited subthreshold or suprathreshold excitatory MP responses to a flash visual stimulus, which were largely dependent on neuronal activity in the visual thalamus but not visual cortex. We further found in an even larger population (~81 %) of ACC neurons that excitatory MP responses could also be evoked by a pain-related electrical stimulus. These findings may reflect potential mechanisms underlying the ACC function that involves information processing of sensory and noxious stimuli.

As a multimodal brain region, ACC has been known to be capable of responding to complex stimulation or tasks, including those related to reward [21–23] and aversive stimuli [7, 24–26] as well as attention [1, 9, 10, 27]. However, ACC neuronal activity in response to sensory stimulation remains to be further clarified. In the present study, we have demonstrated that a large amount (~54 %) of layer II/III ACC neurons showed excitatory MP responses to a brief flash of visual stimulation. The widespread distribution of a sensory response in ACC networks may reflect that summation or coordination of the activities evoked by various sensory stimuli can take place at single neuron levels in ACC, a process that may be critical for ACC functions.

As to a specific ACC function of pain perception, previous studies have examined ACC activity in response to painful stimulation [30, 33–35]. In this study, we have used whole-cell recording to measure MP responses of ACC neurons to a painful stimulus. By applying DC electrical stimuli to hind paws, we found notably that a widespread population (81.25 %) of ACC neurons exhibited excitatory MP responses, with 43.75 % recorded neurons showing SR responses. The widespread distribution of electrical stimulus-elicited MP responses indicates that the majority of the ACC neuron that can respond to a sensory stimulus (as we observed with the flash visual stimulus) is also responsive to a painful stimulus. Thus, in addition to pain perception, the co-existing responsiveness to both sensory and painful stimuli in the same ACC neuron is likely to serve as a neural mechanism of some other ACC functions [1–4], for example associative fear memory.

In the present study, the ACC responses to the flash stimulus have been indicated to originate from the visual thalamus, and were further relayed by the major pathway bypassing the primary visual cortex that determines the response magnitude. Previous studies have demonstrated that the sensory thalamus directly projects to numerous brain regions, including the prefrontal cortex, parietal cortex, temporal cortex, nucleus accumbens, and amygdala [36–41]. Some or all of these regions could be responsible for the relay of visual information to ACC, which bypassed the visual cortex. However, it does not necessarily reflect that the amplitude of ACC responses is unchanged by visual cortical activity in any situation. When the brain receives complex information (e.g., in tasks related to attention, emotion, or learning and memory), visual cortical modulation on response amplitudes could take place. On the other hand, no cell was found to show inhibitory responses to the flash stimulus, suggesting no responses in local interneuronal networks. Likewise, activation of ACC interneurons could occur during the processing of complex information.

Because the magnitudes of flash-evoked ACC responses (including the MP and SR responses, see Fig. 6) were found to be unchanged by visual cortical activity, these visual responses, by themselves, could be of certain physiological functions that are independent on visual perception. As a support of this hypothesis, a phenomenon of ‘blindsight’ has been reported in subjects undergoing visual cortical damage, in which patients can still show some responses to certain visual stimuli (e.g., emotional responses to stimulation containing fear signals) independently from conscious vision [42–45]. In this brain function, ACC could be one of the engaged brain regions, given its contribution to attention and emotion.

As compared with the response magnitudes, the temporal pattern distributions of ACC responses have been found to be susceptible to modulation by visual cortical activity. Thus, the temporal pattern of ACC activity, at single neuron and population levels, may be a particularly important strategy used by ACC to represent sensory information during executing the related tasks.

In summary, we have shown MP and SR responses of layer II/III ACC neurons to both a visual stimulus and pain-related electrical stimulus. Our findings may provide further understanding of the neural mechanism that is used for ACC functions on the basis of sensory information processing.

## Abbreviations

ACC: Anterior cingulate cortex
LGN: Lateral geniculate nucleus
MP: Membrane potential
SR: Spike rate
SRR: Spike-rate response
NSRR: No spike-rate response
PFA: Paraformaldehyde
PSTHs: Peristimulus time histogram
V1: Primary visual cortex

## References

[1] Bryden DW, Johnson EE, Tobia SC, Kashtelyan V, Roesch MR (2011). Attention for learning signals in anterior cingulate cortex. J Neurosci.

[2] Foster K, Orona E, Lambert RW, Gabriel M (1980). Early and late acquisition of discriminative neuronal activity during differential conditioning in rabbits: specificity within the laminae of cingulate cortex and the anteroventral thalamus. J Comp Physiol Psychol.

[3] Frankland PW, Bontempi B, Talton LE, Kaczmarek L, Silva AJ (2004). The involvement of the anterior cingulate cortex in remote contextual fear memory. Science.

[4] Maviel T, Durkin TP, Menzaghi F, Bontempi B (2004). Sites of neocortical reorganization critical for remote spatial memory. Science.

[5] Davis KD, Taylor KS, Hutchison WD, Dostrovsky JO, McAndrews MP, Richter EO, Lozano AM (2005). Human anterior cingulate cortex neurons encode cognitive and emotional demands. J Neurosci.

[6] de Araujo IE, Rolls ET, Velazco MI, Margot C, Cayeux I (2005). Cognitive modulation of olfactory processing. Neuron.

[7] Nili U, Goldberg H, Weizman A, Dudai Y (2010). Fear thou not: activity of frontal and temporal circuits in moments of real-life courage. Neuron.

[8] Takahashi H, Kato M, Matsuura M, Mobbs D, Suhara T, Okubo Y (2009). When your gain is my pain and your pain is my gain: neural correlates of envy and schadenfreude. Science.

[9] Bush G, Luu P, Posner MI (2000). Cognitive and emotional influences in anterior cingulate cortex. Trends Cogn Sci.

[10] Totah NK, Kim YB, Homayoun H, Moghaddam B (2009). Anterior cingulate neurons represent errors and preparatory attention within the same behavioral sequence. J Neurosci.

[11] Bliss TV, Collingridge GL, Kaang BK, Zhuo M (2016). Synaptic plasticity in the anterior cingulate cortex in acute and chronic pain. Nat Rev Neurosci.

[12] Schnitzler A, Ploner M (2000). Neurophysiology and functional neuroanatomy of pain perception. J Clin Neurophysiol.

[13] Price DD (2000). Psychological and neural mechanisms of the affective dimension of pain. Science.

[14] Johansen JP, Fields HL, Manning BH (2001). The affective component of pain in rodents: direct evidence for a contribution of the anterior cingulate cortex. Proc Natl Acad Sci U S A.

[15] Devinsky O, Morrell MJ, Vogt BA (1995). Contributions of anterior cingulate cortex to behaviour. Brain.

[16] Jones BF, Witter MP (2007). Cingulate cortex projections to the parahippocampal region and hippocampal formation in the rat. Hippocampus.

[17] Paus T (2001). Primate anterior cingulate cortex: where motor control, drive and cognition interface. Nat Rev Neurosci.

[18] Stefanacci L, Amaral DG (2000). Topographic organization of cortical inputs to the lateral nucleus of the macaque monkey amygdala: a retrograde tracing study. J Comp Neurol.

[19] Vogt BA, Miller MW (1983). Cortical connections between rat cingulate cortex and visual, motor, and postsubicular cortices. J Comp Neurol.

[20] Vogt BA, Vogt L, Farber NB, Bush G (2005). Architecture and neurocytology of monkey cingulate gyrus. J Comp Neurol.

[21] Amiez C, Joseph JP, Procyk E (2006). Reward encoding in the monkey anterior cingulate cortex. Cereb Cortex.

[22] Korn CW, Prehn K, Park SQ, Walter H, Heekeren HR (2012). Positively biased processing of self-relevant social feedback. J Neurosci.

[23] Williams ZM, Bush G, Rauch SL, Cosgrove GR, Eskandar EN (2004). Human anterior cingulate neurons and the integration of monetary reward with motor responses. Nat Neurosci.

[24] Etkin A, Egner T, Kalisch R (2011). Emotional processing in anterior cingulate and medial prefrontal cortex. Trends Cogn Sci.

[25] Fan J, Gu X, Liu X, Guise KG, Park Y, Martin L, de Marchena A, Tang CY, Minzenberg MJ, Hof PR (2011). Involvement of the anterior cingulate and frontoinsular cortices in rapid processing of salient facial emotional information. Neuroimage.

[26] Li XY, Ko HG, Chen T, Descalzi G, Koga K, Wang H, Kim SS, Shang Y, Kwak C, Park SW (2010). Alleviating neuropathic pain hypersensitivity by inhibiting PKMzeta in the anterior cingulate cortex. Science.

[27] Botvinick MM, Cohen JD, Carter CS (2004). Conflict monitoring and anterior cingulate cortex: an update. Trends Cogn Sci.

[28] Ning L, Ma LQ, Wang ZR, Wang YW (2013). Chronic constriction injury induced long-term changes in spontaneous membrane-potential oscillations in anterior cingulate cortical neurons in vivo. Pain Physician.

[29] Wu LJ, Li X, Chen T, Ren M, Zhuo M (2009). Characterization of intracortical synaptic connections in the mouse anterior cingulate cortex using dual patch clamp recording. Mol Brain.

[30] Koga K, Li X, Chen T, Steenland HW, Descalzi G, Zhuo M (2010). In vivo whole-cell patch-clamp recording of sensory synaptic responses of cingulate pyramidal neurons to noxious mechanical stimuli in adult mice. Mol Pain.

[31] May PJ (2006). The mammalian superior colliculus: laminar structure and connections. Prog Brain Res.

[32] Sillito AM, Cudeiro J, Jones HE (2006). Always returning: feedback and sensory processing in visual cortex and thalamus. Trends Neurosci.

[33] Iwata K, Kamo H, Ogawa A, Tsuboi Y, Noma N, Mitsuhashi Y, Taira M, Koshikawa N, Kitagawa J (2005). Anterior cingulate cortical neuronal activity during perception of noxious thermal stimuli in monkeys. J Neurophysiol.

[34] Peyron R, Laurent B, Garcia-Larrea L (2000). Functional imaging of brain responses to pain. A review and meta-analysis (2000). Neurophysiol Clin.

[35] Wang J, Cao B, Yu TR, Jelfs B, Yan J, Chan RH, Li Y (2015). Theta-frequency phase-locking of single anterior cingulate cortex neurons and synchronization with the medial thalamus are modulated by visceral noxious stimulation in rats. Neuroscience.

[36] Cappe C, Morel A, Barone P, Rouiller EM (2009). The thalamocortical projection systems in primate: an anatomical support for multisensory and sensorimotor interplay. Cereb Cortex.

[37] Jayaraman A (1985). Organization of thalamic projections in the nucleus accumbens and the caudate nucleus in cats and its relation with hippocampal and other subcortical afferents. J Comp Neurol.

[38] LeDoux JE, Farb C, Ruggiero DA (1990). Topographic organization of neurons in the acoustic thalamus that project to the amygdala. J Neurosci.

[39] Linke R, Schwegler H (2000). Convergent and complementary projections of the caudal paralaminar thalamic nuclei to rat temporal and insular cortex. Cereb Cortex.

[40] Schmahmann JD, Pandya DN (1990). Anatomical investigation of projections from thalamus to posterior parietal cortex in the rhesus monkey: a WGA-HRP and fluorescent tracer study. J Comp Neurol.

[41] Xiao D, Zikopoulos B, Barbas H (2009). Laminar and modular organization of prefrontal projections to multiple thalamic nuclei. Neuroscience.

[42] Anders S, Birbaumer N, Sadowski B, Erb M, Mader I, Grodd W, Lotze M (2004). Parietal somatosensory association cortex mediates affective blindsight. Nat Neurosci.

[43] Bertini C, Cecere R, Ladavas E (2013). I am blind, but I “see” fear. Cortex.

[44] Leopold DA (2012). Primary visual cortex: awareness and blindsight. Annu Rev Neurosci.

[45] Stoerig P, Cowey A (1997). Blindsight in man and monkey. Brain.

